# Glycemic control and associated factors among type 2 diabetic patients at Shanan Gibe Hospital, Southwest Ethiopia

**DOI:** 10.1186/s13104-017-2924-y

**Published:** 2017-11-15

**Authors:** Daniel Miteku Yigazu, Tigestu Alemu Desse

**Affiliations:** 1 School of Pharmacy, College of Health Sciences, Jimma University, Jimma, Ethiopia; 2 Department of Clinical Pharmacy and Pharmacology, School of Pharmacy, College of Health Sciences, Addis Ababa University, Addis Ababa, Ethiopia

**Keywords:** Diabetes mellitus, Type two diabetes, Glycemic control, Ethiopia

## Abstract

**Objective:**

The aim of this study was to assess the rate of glycemic control and factors affecting glycemic control in type 2 diabetic patients.

**Results:**

A total of 174 type 2 diabetic patients were interviewed and were studied. Mean age of the patients was 48.98 ± 14.96 years (range 18–80 years). More than half (51.7%) of the patients were males. About a third of patients, 53 (30.5%), were on antidiabetic medications for less than 5 years. The most common prescribed antidiabetic medications were insulin, 48 (27.6%), and metformin 15 (8.6%). One hundred seven (61.5%) patients were on combination therapy (two drug treatment) and the remaining patients were on monotherapy. The majority, 103 (59.2%), of patients had uncontrolled blood glucose. A larger proportion of female patients, 54 (52.4%), had uncontrolled blood glucose than males. Level of education (p < 0.001) and duration of diabetes treatment (p < 0.001) were significantly associated with glycemic control. Adherence of patients to regular follow up (Adjusted Odds Ratio (AOR) = 2.42, 95% CI 1.08–5.44, p = 0.03) and diabetes treatment for 5–10 years (AOR = 4.64, 95% CI 1.79–12.06, p = 0.002) are found to be independent predictors of glycemic control among type 2 diabetes patients.

## Introduction

Diabetes mellitus (DM) is a metabolic disorder that may be caused by multiple etiologies. It is characterized by chronic hyperglycemia due to defects in insulin secretion, insulin action, or both [1]. There are four types of diabetes mellitus: type 1 diabetes, type 2 diabetes, gestational diabetes, and other specific types [2].

Type 2 diabetes constitutes about 85–95% of all diabetes in high-income countries with a higher percentage in low- and middle-income countries due to rapid socio-cultural changes, ageing populations, increasing urbanization, reduced physical activity and unhealthy lifestyle and behavioral patterns [2, 3]. It is a leading cause of blindness, end stage renal disease and stroke. These complications are two to five times more common among diabetic patients [4]. Type 2 diabetes is associated with increased morbidity and mortality compared with the general population [5]. About 80% of diabetes deaths occur in low and middle income countries [6].

Evidence shows that the main therapeutic goal for all diabetes patients is maintaining good glycemic control so as to prevent organ damage and micro-vascular and macro-vascular complications. However, the majority of patients fail to achieve good glycemic control and the reasons for poor glycemic control are complex and multi factorial [7, 8]. Patients with poor glycemic control may experience many symptoms of diabetes, possible cognitive impairment, immune dysfunction, and hospital admissions and complications [6, 9].

In Ethiopia, the prevalence of diabetes admission has increased from 1.9% in 1970 to 9.5% in 1999 of all medical admissions [10] most importantly uncontrolled blood glucose due to non-compliance to antidiabetic medications [11]. As to the authors’ knowledge, studies on the rate of glycemic control in type 2 diabetes patients in Southwest Ethiopia are limited. The aim of this study was to assess the rate of glycemic control and factors affecting glycemic control in type 2 diabetic patients on follow-up at Shanan Gibe Hospital, Southwest Ethiopia.

## Main text

### Research methods and patients

A cross sectional study was conducted at Shanan Gibe Hospital from February to March, 2016. Shanan Gibe Hospital is a public hospital which serves both inpatients and out patients. It is located in Jimma Zone, Oromia region, Southwest Ethiopia. Diabetic patients get follow up service from the hospital twice a week.

Jimma University Institutional Review Board approved the research. We received a letter of permission to access data from clinical director of the hospital. To collect for demographic data, we obtained written informed consent of the study participants prior to interviews. The right of participants to withdraw from an interview at any time was maintained. We determined sample size using single population proportion formula. We studied all type 2 diabetes patients who fulfilled the inclusion criteria and visited the diabetes clinic of the hospital during the study period. The main outcome of the study was glycemic control. We trained data collectors to maintain the quality of the data. We translated an English version of the questionnaire to local language and back translated to English. The data collection tool was also pretested.

We included participants in the study with type 2 diabetes and ≥ 18 years old, patients who were on antidiabetic medication(s) treatment for at least 6 months, patients with at least three consecutive blood glucose measurements for 3 months, and patients who consented to participate. Patients with hearing problems and previously diagnosed psychiatric illness were excluded from the study. Data were collected by two trained pharmacists. We used a pretested structured questionnaire to collect data about sociodemographic characteristic. Data abstraction format was used to collect data on clinical characteristics of patients such as diagnosis, duration of illness, dosage regimen of medications, comorbidities, diabetes complications, and blood glucose measurements. A structured questionnaire was used to collect patients’ demographics.

### Statistical analysis

We analyzed the data using SPSS Version 21.0 (Chicago, SPSS Inc.). We used Chi square tests to see the association between categorical variables and level of blood glucose control. To examine factors affecting medication adherence, we performed a multivariate logistic regression analysis. Variables with p < 0.25 on a univariate logistic regression analysis were entered into a multivariate logistic regression analysis model to identify independent factors that affect glycemic control. Variables with p < 0.05 were considered statistically significant with 95% level of confidence.

### Operational definitions and definition of terms

Controlled blood glucose was defined as average fasting blood glucose measurement 80–130 mg/dL [12]. Uncontrolled blood glucose was defined as patients whose average blood glucose measurements on three consecutive visits is > 130 or < 70 mg/dL. Family adherence support was defined if patients get any kind of support and/or advice from their family member related to medication (s) and life style conditions.

## Results

### Baseline characteristics of patients

We studied 174 patients who visited the hospital during the study period. More than half, 90 (51.7%), of the patients were males. Mean age of the patients was 49.98 ± 14.9 years (range 18–80 years). More than a third of the patients, 74 (42.5%), attended primary school. Seventy-four (42.5%) patients did not adhere to their regular follow up at the diabetes clinic of the hospital. The majority of patients, 111 (63.8%), did not get adherence support from their families (Table 1). About a third of patients, 53 (30.5%), were on antidiabetic medications for less than 5 years. Almost half of the patients were on treatment for more than 10 years 86 (49.4%). Ninety (51.7%) patients had at least one co-morbidity. Hypertension was the major type of co-morbidity; 71 (78.9%). Seventy-one (40.8%) of the patients had ≥ 1 diabetes related complication. Diabetic neuropathy was the most common complication; 31 (43.7%).

**Table 1:** Characteristics of type 2 diabetes patients at Shanan Gibe Hospital, Southwest Ethiopia

Variable category	Characteristics	n (%)
Sex	Male	90 (51.7)
	Female	84 (48.3)
Age category	< 30	21 (12.1)
	30–60	114 (65.5)
	> 60	39 (22.4)
Level of educational	Illiterate	60 (34.5)
	Primary education	74 (42.5)
	Secondary education	35 (20.1)
	College and above	5 (2.9)
Marital status	Single	14 (8.0)
	Married	142 (81.6)
	Divorced/separated	18 (10.3)
Regular follow up	No	74 (42.5)
	Yes	100 (57.5)
Family support	Yes	63 (36.2)
	No	111 (63.8)
Duration of diabetes treatment (years)	< 5	53 (30.5)
	5–10	35 (20.1)
	> 10	86 (49.4)
Co-morbidity	Yes	90 (51.7)
	No	84 (48.3)
Type of co-morbidity	Hypertension	71 (78.9)
	Ischemic heart disease (IHD)	10 (11.1)
	Hypertension + IHD	5 (5.6)
	CKD	2 (2.2)
	Others	2 (2.2)
Diabetic complications	Yes	71 (40.8)
	No	103 (59.2)
Type of diabetic complication	Neuropathy	31 (43.7)
	Retinopathy	10 (14.1)
	Retinopathy + Neuropathy	28 (39.4)
	Retinopthy + Neuropathy + Nephropathy	2 (2.8)

*Others* dyslipidemia and obesity

*CKD* chronic kidney disease, *IHD* Ischemic heart disease

The most common prescribed antidiabetic medication was insulin, 48 (27.6%) followed by metformin 15 (8.6%). More than half; 92 (52.9%) of the patients were taking a combination of metformin and glibenclamide (Table 2). One hundred seven (61.5%) of patients were on combination therapy (two drug treatment) and the remaining patients were on monotherapy. Sixty-two (35.6%) patients had concomitant medication(s) for the treatment of comorbidities. Enalapril was the most common prescribed concomitant medication; 47 (75.8%).

**Table 2:** Type of antidiabetic medications of type 2 diabetic patients attending the diabetic clinic of Shaman Gibe hospital

Variable category	Type of antidiabetic medication	n (%)
Type of antidiabetic medication	Insulin	48 (27.6)
	Metformin	15 (8.6)
	Glibenclamide	4 (2.3)
	Insulin + metformin	15 (8.6)
	Metformin + glibenclamide	92 (52.9)
Concomitant medications	Yes	62 (35.6)
	No	112 (64.4)
Type of concomitant medication	Enalapril	47 (75.8)
	Enalapril + ASA + atenolol	6 (9.7)
	Enalapril + ASA	3 (4.8)
	Enalapril + ASA + hydrochlorothiazide	3 (4.8)
	Others	3 (4.8)

*Others* include metoprolol, amlodipine and atorvastatin

*ASA* acetyl salicylic acid

### Glycemic control and factors affecting glycemic control

Fasting blood glucose readings of last three clinic visits were obtained from patients’ medical records and the mean last three fasting blood glucose measurements were used to determine the level of glycemic control. Mean fasting blood glucose measured over 3 months was 130.3 ± 30.7 mg/dL. The minimum and maximum recorded fasting blood glucose measurements were 33 and 254 mg/dL respectively. Less than half, 71 (40.8%), of the patients achieved the American Diabetes Association recommended target fasting blood glucose range (80–130 mg/dL). The majority, 103 (59.2), of patients had uncontrolled blood glucose. The rate of glycemic control was 71 (40.8%).

A larger proportion of female patients, 54 (52.4%), had uncontrolled blood glucose than males. Level of education (p < 0.001) and duration of diabetes treatment (p < 0.001) were significantly associated with glycemic control.

On a multivariable logistic regression analysis (Table 3), adherence of patients to regular follow up (adjusted odds ratio (AOR) = 2.42, 95% CI 1.08–5.44, p = 0.03) and diabetes treatment for 5–10 years (AOR = 4.64, 95% CI 1.79–12.06, p = 0.002) were found to be independent predictors of glycemic control among type 2 diabetes patients at Shanan Gibe Hospital.

**Table 3:** Factors associated with glycemic control of type 2 diabetes patients at Shanan Gibe Hospital, Southwest Ethiopia

Variable category		Glycemic control		AOR (95% CI)
		Controlled (n)	Uncontrolled (n)	
Gender	Male	41	49	1.58 (0.79–3.15)
	Female	34	54	1
Educational status	Illiterate	10	50	1
	Primary school	39	35	2.20 (0.81–5.97)
	Secondary school	19	16	2.31 (0.71–7.52)
	College and above	3	2	1.79 (0.20–15.86)
Regular follow up	No	17	57	1
	Yes	54	46	2.42 (1.08–5.44)*
Duration of treatment (years)	< 5	28	25	2.03 (0.85–4.84)
	5–10	23	12	4.64 (1.79–12.06)*
	> 10	20	66	1

*AOR* adjusted odds ratio

p < 0.05 was considered statistically significant

* Statistically significant

## Discussion

This study assessed the magnitude of glycemic control and factors affecting glycemic control among type 2 diabetic patients at Shanan Gibe Hospital, Southwest Ethiopia. The mean fasting blood glucose was 130.3 ± 30.7 mg/dL. We found that the majority of patients had poor glycemic control. Adherences to regular follow up schedule and diabetes treatment for 5–10 years were predictors of glycemic control.

In our study, the mean fasting blood glucose over 3 months was 130.3 ± 30.7 mg/dL. This value is lower than the study in Malaysia [13] where the mean fasting blood glucose was 166.5 ± 86.4 mg/dL. It was also higher than the studies in Addis Ababa (190 ± 89.6 mg/dL) [14] and Jimma university specialized hospital (171 ± 63 mg/dL) [15], and higher than the American Diabetic Association recommendation [12]. This higher value indicates that the rate of blood glucose in our setup is poor and does not meet the recommended target of the American Diabetes Association. This may be due to poor medication adherence, poor lifestyle conditions and, failure to adhere to regular follow up at diabetes clinic.

We found that the majority of our patients (59.2%) had uncontrolled blood glucose. The rate of uncontrolled blood glucose in our study finding is lower than the findings in Jordan [16], Malaysia [13] and India [17] ranging from 65.1 to 78.6%. In our study, we measured fasting blood glucose to assess glycemic control whereas in the for stated studies, glycated hemoglobin (HbA1C) was measured to assess the level of glycemic control.

The reason for the difference in the rate of glycemic control between our study and other studies may be the variation in clinical characteristics of the participants. For example, in Malay study, the participants were older than ours and the patients with duration of diabetes < 5 years in ours was 30.5% whereas in Malay it was 24.2%. In Jordan, about 50% of the patients had diabetes duration > 7 years and the majority of patients had obesity, dyslipidemia and hypertension. In addition, in Indian study, larger proportion of patients were > 50 years old. Evidence also shows that longer duration of diabetes, use of multiple medications, and old age are associated with poorly controlled blood glucose [13, 16, 18, 19].

Other studies in Ethiopia showed that the rate of uncontrolled blood glucose ranged from 48.7% based on HBA1C measurement to 70.9% on fasting blood glucose measurement [20, 21]. This is comparable to our study finding with a slightly higher value (70.9%) in only one study. The similarity of our study finding with other local studies may be due to similar characteristics of the study patients and similar diabetes management practice.

The rate of uncontrolled blood glucose in our study finding was higher than the study findings in Nigeria [22], China [23], Brazil [24], Mexico [25] and the United States [26]. Level of uncontrolled blood glucose in these countries ranged from 12.9 to 57%. This variation could be due to differences in patient characteristics and differences in diabetes management practices. For example, in our study, the rate of illiteracy was high and regular follow up of diabetes patients was minimal. In addition, appropriate diabetes management guideline was used in other studies while no diabetes management guideline was used in the management of type 2 diabetes in our hospital. Moreover, fasting blood glucose was measured to assess the level of glycemic control in our setup while glycated hemoglobin was used in the studies we compared.

In our study, the number of illiterate patients with uncontrolled blood glucose was high and about half (48.3%) of them had uncontrolled blood glucose. Education level was also significantly associated with glycemic control in our study. The possible explanation could be illiterate patients may have low diabetes knowledge, low self-management behaviors, lower self-efficacy and lower continuity of care leading to poor glycemic control. However, in contrast to our study finding, in the United Kingdom, patients with lower level of educational had better compliance to medications and more trust in the physicians’ advice [27].

The duration of diabetes mellitus was significantly associated with glycemic control. A study in Hong Kong [26] revealed that patients with longer duration of diabetes and more complex treatment regimens were associated with poorer glycemic control. Juarez et al. [28] also reported that patients who had had diabetes for 10 years were about nine times more likely to have poor glycemic control than those who had had diabetes for 3 years. A longer duration of diabetes negatively affects glycemic control, possibly because of progressive impairment of insulin secretion over time as a result of β‐cell failure. Therefore, as the disease progresses, most patients require an increase in their pharmacotherapy to maintain glycemic control.

Adherence of patients to regular follow up and diabetes treatment for 5–10 years were found to be independent predictors of glycemic control among type 2 diabetes patients. This study finding is similar to other studies [13, 18, 19]. Viana et al. [29] and Ramirez et al. [25] also reported that duration of diabetes, use of insulin, and unsatisfactory patient physician relationship were significantly associated with level of glycemic control.

## Conclusion

In conclusion, our study finding showed that the rate of poor glycemic control was high. Level of education and duration of diabetes treatment were significantly associated with glycemic control. A longer duration of diabetes and lack of regular follow up at diabetes clinic independently affect the rate of glycemic control in type 2 diabetes patients. Therefore, we recommend that Shanan Gibe Hospital develop strategies for improving glycemic control of type 2 diabetes patients.

## Limitations

The study has some limitations.

- The sample size was relatively small which may limit generalization of the study findings to a larger population of type 2 diabetes patients.
- We used fasting blood glucose to assess level of glycemic control as there was no laboratory facility to measure glycated hemoglobin. Measurement of glycated hemoglobin would show the rate of glycemic control over 3 months while fasting blood sugar may have some drawbacks to show the true level of glycemic control.
